# Detroit Academy of Medicine

**Published:** 1879-09

**Authors:** 


### DETROIT ACADEMY OF MEDICINE.

May 27, 1879.

Dr. McGraw read a detailed report, related by Freund in a German journal, of a case of extirpation of the uterus, in which both ureters were tied. The patient lived almost four days and never presented any brain symptoms till the afternoon of her death. There was during this time some nausea. slight emesis, profuse salivation, and absolute absence of urine In the post-mortem examination, the ureters were found securely tied and dilated on the proximal side, to the size of the middle finger.

### PATHOLOGICAL SPECIMENS.

Dr. Carstens exhibited a foot he had amputated from a girl the same day. An operation of necrosis of the tarsal bones commenced, but the latter were found to be so totally destroyed that removal of the entire foot and a portion of the tibia and fibula was necessary.

Dr. Noyes—I would like to show to the society two eyes which I enucleated the last week.

The first is from a gentleman of Saginaw, aged 43, who was hit in the left eye by a nail, ten years ago. After the first effects of the injury passed off he recovered perfect sight, and it was only five years after that he noticed some difficulty of vision when looking directly forwards. Finally vision was entirely lost. During all this time there had been no signs of inflammation. Last February he experienced the first pain in the eye since the accident, and his sufferings were so great that he was obliged to take to his bed for four weeks in a darkened room. His physicians did all they could to help him, bnt still he found no entire relief. Last Wednesday he came to me, and I found the eye greatly contracted at the sclero-corneal junction, pupil dilated and showing a dark reflex from the fundus. The right eye was now sympathetically inflamed, and I accordingly advised immediate removal of the left. On cutting open the eye, I found this tumor at the posterior pole. The retina was completely detached and formed a bridge across the interior of the eye. The tumor at first was very black, and I believe it to be a melano-sarcoma. This is the third which I have seen, and Knapp mentions only eleven in his work, so that they are certainly rare. If the characteristic growth of the tumor extends outside of the globe, the death of the patient is assured. Had I been aware of the presence of this tumor, I would have snipped the optic nerve as close to the foramen as possible.

The second eye was taken from a young physician who lost it last fall from the effects of a gonorrhœal ophthalmia, caught from one of his patiens. Lately the sound eye had commenced to trouble him, and I advised the immediate removal of the the injured one. He rather hesitated about the operation, but in three days such a change had taken place that he begged for speedy relief. The eye was removed and its interior was found filled with lymph, which, pressing on the ciliary nerves, had probably caused the great pain which he had experienced.

Once sympathetic trouble has truly commenced in the uninjured eye, *i. e.*, inflammation, etc., it is generally too late to be of much service ; such was the condition of this patient, but since the operation the other eye has decidedly improved.

Dr. Walker exhibited a specimen of partial calcareous degeneration of a sebaceous cyst.

PREVAILING DISEASES.

D. Johnson reported that he had met with a number of cases of follicular pharyngitis.

VERBAL COMMUNICATIONS.

Dr. Walker—Three weeks ago a child, nineteen months old, whilst playing, swallowed a bean. The mother found the child in a suffocating condition, but by shaking it energetically, relieved it. Several physicians were called, but the child was so comfortable that they did not think it advisable to do anything. Soon, however, other attacks of dyspnœa came on, so that Sunday the child was operated upon, but nothing was found. The following Friday the child suddenly died. In the autopsy, the bean was found just above the bifurcation of the trachea.

Dr. McGraw—In no class of cases is it more difficult to decide upon our plan of action than in the case of foreign bodies in the trachea. It is a serious question whether we shall operate or not. I recall the case of a child who was said to have swallowed a ten-penny nail. She seemed to be perfectly easy, and after careful auscultation I could not detect its presence. After a sudden fit of coughing and strangling one day, up came the nail. Such cases frequently occur, and if the foreign body is found we expect a cure.

Dr. Carstens reported that his case of strangulated hernia spoken of in the last meeting had completely recovered from the operation.

Adjourned.

E. A. CHAPOTON, Secretary.

---

June 10, 1879.

Dr. Ward read his inaugural paper on the subject of Physiological Antagonism, the Therapeutic Law.

PATHOLOGICAL SPECIMENS.

Dr. Andrews—During the past week I was called to a case of labor, where I obtained this specimen. The patient was a woman aged 37 years, who, during eleven years of wedded life had borne ten children, four of whom, however, were twins. The third labor since was peculiar in this respect, that one twin was born twelve hours after the first. No particular history of the delivery of the placentæ of these children can be obtained. The labor preceding the present one was normal in every respect. In the latter case the presentation was an elbow one, but by manipulation the vertex was made to enter the canal. A small but normal placenta was removed from the vagina, but immediately following it was felt another mass of cartilaginous consistency, and presenting the elevations and depressions of the normal uterine wall. I at first thought that it was a portion of the womb, but on careful examination of the uterus and vagina I found them intact, and then recognized that the mass was another placenta. The most searching care failed to find the slightest traces of a blighted ovum, so that I do not consider a twin conception probable in this instance. Was this placenta retained from the pregnancy before the last?

Dr. Jenks—This is certainly an anomalous case, and reminds me of only one in my experience. On that occasion I had removed what I supposed to be a uterine tumor, of about one-quarter of the size of this afterbirth. On examination, however, I discovered it to be of placental origin, and then on questioning the patient I found that she had not been pregnant for three years.

Dr. Carsten—Does not the appearance of this mass resemble the placenta of a fœtus dying early in pregnancy, whilst another child went on to full term? To my eyes the similarity is most striking, and I cannot but believe that the other fœtus was either lost or absorbed, and that this growth is not the product of a previous pregnancy.

Dr. Carrier—Dr. Carstens' suggestions remind me of a case in which the order of events was exactly as stated. I was called to a woman who was having uterine pains and some sanguineous discharge, and shortly after my arrival she gave birth to a fœtus about one and a half inches long. Six months afterwards she was delivered of a full grown child, so that the abortion of the first seemed to have no effect upon the second ovum.

D. Cleland—Some time since I attended a lady who for several weeks, presented symptoms indicating the presence of a dead fœtus in her uterus. Suddenly one night the membranes placenta, waters and fœtus were expelled intact. The doctor exhibited his specimen.

Dr. Inglis—In attending to a case of obstetrics to which I had been called, I found that a peculiar and certainly a rare accident had taken place. The conception had been a double one, but flattened against the full-term fœtus was found a dead one of about four months growth. The full-term fœtus was likewise dead, and herein lies the singularity of the case, that its death was caused by strangulation of its cord by that of the blighted ovum only a short time before normal delivery would have taken place.

Dr. Connor—To return to the subject brought up by the pathological specimen, I do not think it probable that this placenta can be referred to the third last pregnancy. When one takes its size into consideration, as well as that of the interior of the uterus when contracted down, it seems as if the spermatozoa would have had a rather slim chance of causing conception to take place, if such a body had remained in the womb.

PREVAILING DISEASES.

Dr. Ranney reported some scarlatina and measles as prevailing in and around Lansing.

Dr. Cleland had met scarlatina, measles, and a few cases of mumps.

Dr. Hawes had had some cases of catarrhal pharyngitis.

Dr. Carstens had also seen some cases of erysipelas.

Dr. Inglis was meeting with the whooping-cough and ague.

VERBAL COMMUNICATIONS.

Dr. Hawes—Last Sunday afternoon I examined, in the presence of a number of medical gentlemen, the remains of a patient who had died the day before from some puerperal accident. The lungs and heart were in a fairly normal condition ; not the slightest inflamation of the peritoneum was found ; the internal abdominal organs were congested and slightly enlarged, but otherwise normal ; near the fundus uteri was found a portion of the placenta in a breaking down condition, and the absorption of this matter was judged to be the primal cause of death.

Dr. Gilbert—This woman was one of a number of cases of puerperal trouble which we have lately had in the Women's Hospital. The first patient was a farmer's wife, a perfect Amazon in appearance and strength. Within seventy-two hours of her delivery she was dead. The case mentioned by Dr. Hawes was about to depart, when, two weeks after giving birth to the child, she had a severe chill during the night, and in the morning was found with a temperature of 106°. Large doses of quinine controlled the fever, but on the sixth day she also died. The third case is now convalescing. The second fatal case reminded me of a patient who was confined some years ago in Harper Hospital. She made a very good recovery, but after several days she suddenly began to vomit, and shortly after died. The post-mortem examination revealed nothing that would account for her death.

Dr. Connor—I believe that an explanation of some of the cases of sudden death after labor is the spontaneous coagulation of the blood. To the theory of thrombi had always been objected the impossibility of their passage through the capillaries of the lung, and yet these formations were found in the course of the cephalic circulation. Schmidt has demonstrated that coagulation takes place only when paraglobuline and hæmoglobuline come together. But what causes them to unite? That was the question ; and he answered it last year, when he discovered that in the decomposition of white blood corpuscles a substance was set free which immediately causes them to run together. With such an explanation, we can easily account for some of these sudden deaths, for white cells are suddenly very numerous in the uterus after labor, and as hardly anything can be considered a barrier to their progress, we can imagine them giving out anywhere this substance which causes coagulation. Some of these thrombi may enter the vessels going to the respiratory or cardiac centres, and thus shutting off the circulation, cause an almost instant death.

Dr. Ranney—Last February, whilst attending a lady, I was consulted in reference to her young daughter, a very large child of five years of age. I remarked on looking at her, that her form was rounded out, and that she manifested the reserve of a girl during her forming years. Her mother informed me that the child's breasts, when she was two years old, suddenly commenced to develop, that hair grew upon the pubes, and menstration set in. The menses continued normal for one year, and then changed slightly in color and quantity. An aunt on the father's side commenced menstruation at eleven. The only other case I know in which the menses appeared so early in life, is the one reported by Dr. Marcy, of Massachusetts, two years ago, before the American Medical Association.

Dr. Jenks remarked that he had seen two years ago, in Chicago, a child two and a half years of age whose body above the pelvis was that of a perfect but miniature woman, while the limbs were small and child-like. She menstruated regularly.

Dr. Jenks.—Mr. President, if you will permit me, I should like to return to the subject brought up by the report of Dr. Hawes. I believe the post mortem examination showed a clear case of death from puerperal septicæmia. There was enough placenta left in that uterus to account for several deaths. But the point I wish to bring up is the treatment. In these cases, warm intra-uterine injections are the thing, and they must be insisted upon as long as any rise in temperature denotes blood poisoning. The danger of regurgitation into the peritoneum is a minimum one, for the condition of things is essentially different from that existing in a small uterus. In fact, I now make it a rule of practice to have the vagina flooded out in all my obstetrical cases, and I believe my patients, in consequence, feel more comfortable, and get along better than formerly.

Dr. Andrews of Romeo—I recall a case which occurred many years ago, which showed how a small piece of decomposing material can cause septicæmia. A boy had cut a small flap on one of his toes. Neither the bone nor the nail was implicated. The patient tied the piece in place, and some days afterwards I was called to him as he was suffering from high fever and chills. Seeing the bandaged toe, I removed the dressing, and found the flap in a gangrenous condition. I cut it off, and the boy immediately recovered.

Dr. Chapoton reported that two weeks ago he had delivered a woman at full term of an encephalic fœtus. Bony formation was lacking from the supraorbital processes to the occipital protuberance. As the child lived for twelve hours, the parents were unwilling to give up the specimen.

Dr. Connor reported that a woman had been delivered a few days before at St. Mary's hospital of a fœtus of six months. The tiny creature was still living, and seemed to possess considerable vitality.

E. A. Chapoton, Secretary.

—*Detroit Lancet.*

